# Supplementary material for: Venomix: a simple bioinformatic pipeline for identifying and characterizing toxin gene candidates from transcriptomic data
Source: PeerJ. 2018 Jul 31;6:e5361. doi: 10.7717/peerj.5361 (PMC6074769; doi:10.7717/peerj.5361)
Supplement: Supplemental Information 3 [file peerj-06-5361-s003.gz › FinalOutput_E-6/Toxins/Snake_venom_metalloproteinase_bothrojaractivase_1/finaltree.pdf]

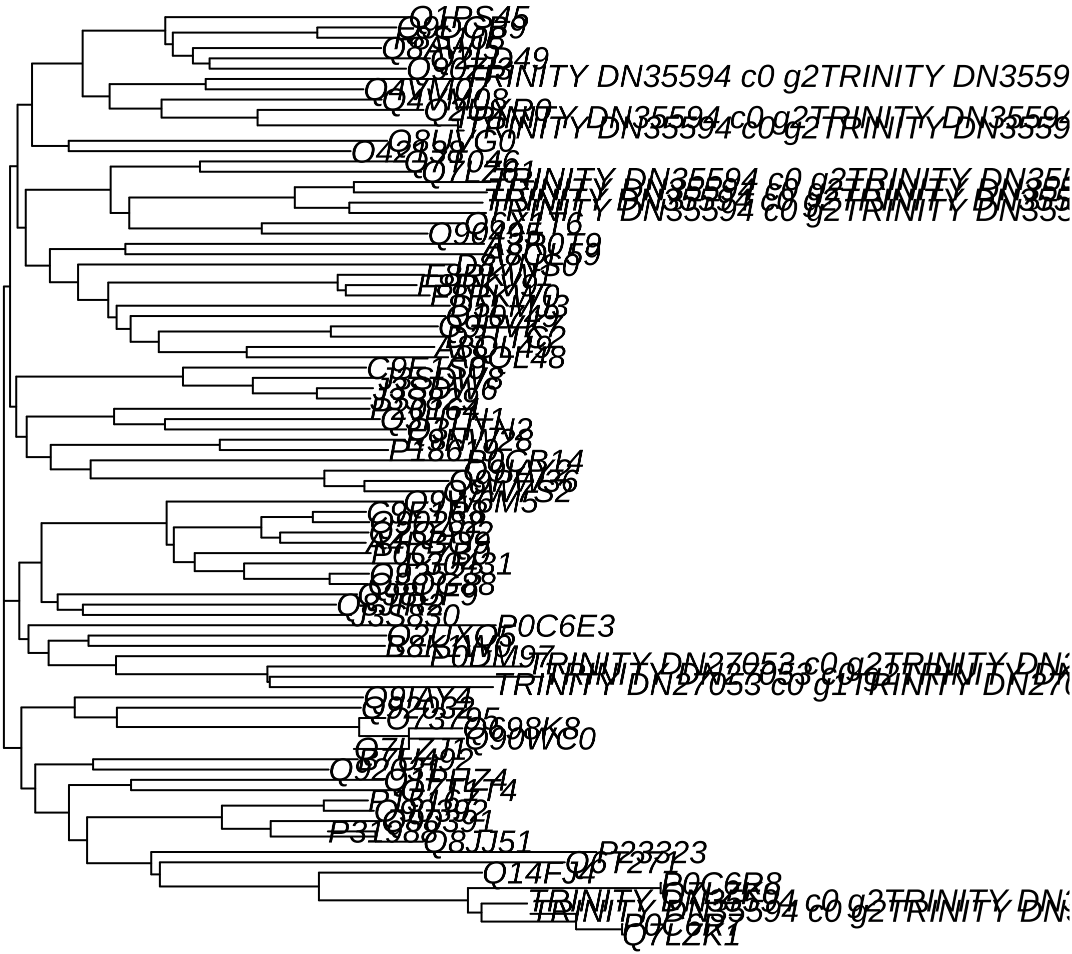
Q1PS45
P0C6E3
Q90WC0
Q8JJ51
P23323
Q14FJ4
Q7L2K1
P31988
Q9IAY4
